# An Energy Efficient Design of Computation Offloading Enabled by UAV

**DOI:** 10.3390/s20123363

**Published:** 2020-06-13

**Authors:** Linpei Li, Xiangming Wen, Zhaoming Lu, Wenpeng Jing

**Affiliations:** 1 School of Information and Communication Engineering, Beijing University of Posts and Telecommunications, Beijing 100876, China; lilinpei@bupt.edu.cn (L.L.); xiangmw@bupt.edu.cn (X.W.); jingwenpeng@bupt.edu.cn (W.J.); 2 Beijing Key Laboratory of Network System Architecture and Convergence, Beijing University of Posts and Telecommunications, Beijing 100876, China; 3 Beijing Laboratory of Advanced Information Networks, Beijing University of Posts and Telecommunications, Beijing 100876, China

**Keywords:** unmanned aerial vehicle, mobile edge computing, offloading, energy efficiency

## Abstract

The data volume is exploding due to various newly-developing applications that call for stringent communication requirements towards 5th generation wireless systems. Fortunately, mobile edge computing makes it possible to relieve the heavy computation pressure of ground users and decrease the latency and energy consumption. What is more, the unmanned aerial vehicle has the advantages of agility and easy deployment, which gives the unmanned aerial vehicle enabled mobile edge computing system opportunities to fly towards areas with communication demand, such as hotspot areas. However, the limited endurance time of unmanned aerial vehicle affects the performance of mobile edge computing services, which results in the incomplete mobile edge computing services under the time limit. Consequently, this paper concerns the energy-efficient scheme design of the unmanned aerial vehicle while providing high-quality offloading services for ground users, particularly in the regions where the ground communication infrastructures are overloaded or damaged after natural disasters. Firstly, the model of energy-efficient design of the unmanned aerial vehicle is set up taking the constraints of the energy limitation of the unmanned aerial vehicle, the data causality, and the speed of the unmanned aerial vehicle into account. Subsequently, aiming at maximizing the energy efficiency of the unmanned aerial vehicle in the unmanned aerial vehicle enabled mobile edge computing system, the bits allocation in each time slot and the trajectory of the unmanned aerial vehicle are jointly optimized. Secondly, a successive convex approximation based alternating algorithm is brought forward to deal with the non-convex energy efficiency maximization problem. Finally, it is proved that the proposed energy efficient scheme design of the unmanned aerial vehicle is superior to other benchmark schemes by the simulation results. Besides, how the performance of proposed scheme design change under different parameters is discussed.

## 1. Introduction

The number of mobile users has been proliferating at a surprising speed lately. With the predication of Cisco, there will be 12.3 billion mobile devices by 2022 [1]. It is also estimated by Cisco that the mobile data traffic is predicted to increase to 77 exabytes every month by 2022, which is a seven-fold growth over 2017 [1]. The conception of cloud computing is presented [2,3] to handle the growing data traffic. The limitations of resources and computing capability for mobile users are offset by the computing power of the cloud. Despite the computing resources that the cloud computing can provide, the computation needs for edge users are not yet meet due to the long distance from the cloud facilities to the edge users. Hence, the longer transmitting time delay and more energy consumption by communication is aroused [4,5]. In addition, the traffic of emerging applications, like virtual reality traffic, augmented reality traffic, and high-definition video traffic, are expected to grow enormously by 2022 [6]. The popularity of such applications that call for intensive computation and strict delay has aggravated the stress on the cloud computing network and cell-edge users. Although the mobile users are equipped with more and more powerful central processing unit (CPU), the need of computation resources and latency are still not met [4]. Thanks to the idea of mobile edge computing (MEC), the burden of the communication network and edge users are alleviated [7,8]. MEC offers cloud-computing capabilities for the edge of the mobile network, within the Radio Access Network (RAN), and in close proximity to mobile users [9].

Nevertheless, the function of ground communication facilities might be not available in some specific areas when the number of users are too large or after disasters. After the ground communication infrastructures are overloaded or destroyed, the communication and computing resources are not sufficient or cannot be provided. Fortunately, the unmanned aerial vehicles (UAVs) have the advantages of easy deployment, easy line-of-sight (LoS) links establishment, and flexible movement, which makes it possible to make use of the UAVs as communication platforms [10,11]. UAVs have lots of compelling applications, owing to the above-mentioned advantages [12,13], such as the delivery of goods [14,15], public safety [16,17], search and rescue missions [18,19,20], and wireless communication platforms [10,21]. In all application fields, utilizing the UAVs to provide wireless links and computing services is one of the prospects towards the upcoming 5th generation wireless systems (5G) [10]. UAVs are able to fly to the designated areas to serve the users in need of urgent communication, thanks to the superiority of high agility, dynamic deployment, and easy LoS links connections establishment. Mounted with MEC equipments, the UAVs have the ability to provide on-demand communication and computation services for users in some specific areas when the fixed infrastructures are not available. The UAVs can be broadly categorized into two types: fixed-wing UAVs and rotary-wing UAVs [21]. The fixed-wing UAVs can move fast, but they have to move towards a certain direction and cannot stay at a fixed point. By comparison, the rotary-wing UAVs can fly in any directions and keep at a certain point. Additionally, how to select the appropriate UAVs as communication platforms is on the basis of the communication scenarios and requirements. In this paper, the UAV-enabled MEC is requested to move flexibly in accordance with the user distribution and the computation need of each user. Thus, rotary-wing UAV is the selection of UAV in this paper to provide offloading services.

Even though the UAV-enabled MEC systems have lots of merits, some issues still need to be overcome. The limitation of battery storage capacity of the UAV is one of the urgent issues [21,22]. Although the battery technology of the UAV is developing, the duration of flight is still finite. Besides, the duration of flight for the UAV is influenced by the payload of the MEC equipments. What is more, the energy consumption that is consumed by communication and computation also reduces the endurance of the UAV in the UAV-enabled MEC system [23]. Consequently, it is important to devise an energy-efficient scheme of the UAV while providing offloading services. Additionally, the limitation of endurance is particularly critical when using the rotary-wing UAV. It is because the small size of the rotary-wing UAVs leads to the limitation of the battery capacity. Hence, we focus on the energy efficiency maximization problem of the UAV while providing offloading services.

### 1.1. Related Work

The idea of cloudlet in mobile computing was first introduced as a trusted, resource-rich computer or cluster of computers that is well-connected to the Internet and available for user by nearby mobile devices to cope with the long latency of cloud computing in 2009 [24]. Furthermore, in 2014, the concept of MEC was first brought forward by European Telecommunications Standards Institute (ETSI) [25].

MEC has garnered lots of attention in the world as one of the methods to tackle the problems of limited computational and storage resources that are caused by cloud computing. In certain areas, like the remote regions, replacing the batteries of devices is difficult and costs a lot. Hence, the problem of reducing the energy consumption of mobile users to extend the lifetime is important while setting up an energy efficient MEC system. In [26], the authors studied the joint optimization problem of radio and computational resources, aiming at minimizing the energy consumption of mobile users in a multi-cell mobile edge-computing scenario. In [27], the authors set two system goals while investigating partial computation offloading: the energy consumption of mobile devices and latency of application execution minimization. The computational speed of smart mobile device (SMD), transmit power of SMD, and offloading ratio were jointly optimized with the two goals. In [28], the authors studied the joint optimization problem of task caching locally and offloading on edge cloud with the objective of minimizing the total energy consumption of mobile devices under the constraints of computing and storage resources. In [29], the authors studied a novel resource allocation approach over both communication and computation resources. Additionally, the data collection in the uplink, computing at the edge, and data delivery in the downlink, were leveraged to minimize the mobile sum-energy that is required for offloading across all users. In [30], the authors investigated the resource allocation for a multiuser mobile-edge computation offloading system based on time-division multiple access and orthogonal frequency-division multiple access, aiming at minimizing the weighted sum mobile energy consumption. In [31], the authors investigated energy-efficient offloading over multiple fading blocks with random channel gains targeting at minimizing the total expected energy consumption of the mobile device. In [32], the energy consumption of smart mobile devices minimization problem was studied under by jointly optimizing the offloading selection, radio resource allocation, and computational resource allocation coordinately. In [33], the authors provided an optimal strategy to associate mobile users to access point and MEC hosts with the objective of minimizing the overall user transmit power under latency constraints.

Although MEC has created opportunities to cope with the computational stress of edge users, the fixed communication infrastructures are not able to provide adequate resources in some scenarios, such as hotspot areas or when the facilities are destroyed by the disasters. UAVs have the advantages of high agility and easy placement, which gives the UAVs the opportunities to provide on-demand communication services hat are mounted with communication equipment. The study of UAV-assisted communication platforms are attracting considerable interests. Firstly, the appropriate placement of the UAV to provide effective coverage needs to be investigated. In [34], the optimal three dimensional (3D) placement of the UAV base station with the goal of maximizing the number of covered users with different Quality-of-Service (QoS) requirements was investigated. In [35], the minimal number of UAVs which ensures that each ground users is covered by at least one UAV was derived. Secondly, the limitation of the battery storage caused by the small size of the UAV affects the duration of communication services offered by the UAV. Hence, it is crucial to design energy efficient algorithms of the UAV while providing communication services under the limited time. In [22], considering both the communication throughput and the energy consumption of the UAV, a simple circular UAV trajectory was optimized to serve a ground terminal aiming at maximizing the energy efficiency of the UAV.

Thanks to the advantages of the UAV, UAV mounted with MEC server can respond to the communication and computation demand quickly. UAV-enabled MEC system has aroused lots of attention in recent years. In [36], the UAV trajectory, the ratio of offloading tasks, and the user scheduling variables were jointly optimized with the goal of minimizing the sum of the maximum delay of all users in each time slot. In [37], the authors studied the weighted sum energy consumptions minimization problem in an UAV-assisted MEC architecture where a UAV serves as a computing server or acts as a relay for further offloading the tasks of the users to the access point. Additionally, the computation resources scheduling, bandwidth allocation, and the trajectory design of the UAV were jointly optimized. In [38], the authors studied the computation rate maximization problems in a UAV-enabled MEC wireless powered system under both partial and binary computation offloading. In [39], the authors studied computing energy consumption minimization problem between the Internet of Things mobile devices and the UAV by jointly optimizing task offloading decision-making, bit allocation during transmission, and the trajectory of the UAV. In [40], the authors studied the minimization problem of the total mobile energy consumption in a UAV-based mobile cloud computing system. The bits allocation and trajectory of the cloudlet were jointly optimized with orthogonal and non-orthogonal multiple access schemes. In [41], the authors studied joint design of computation offloading and resource allocation as well as UAV trajectory for minimization of energy consumption and completion time of the UAV in the UAV-enabled MEC system for Internet of Things. In [42], the authors studied the energy reduction problem in UAV-enabled edge by smartly making offloading decisions, allocating transmitted bits in both uplink and downlink, as well as designing UAV trajectory. In [43], the authors studied the optimization problem to minimize the total required energy of UAV by jointly optimizing the CPU frequencies, the offloading amount, the transmit power, and the UAV trajectory in UAV-enabled wireless powered cooperative MEC system. In [44], an innovative UAV-enabled MEC system was proposed, aiming at minimizing the weighted sum of the service delay of all IoT devices and UAV energy consumption by jointly optimizing UAV position, communication and computing resource allocation, and task splitting decisions. In our previous work [45], we focused on the minimization of the total energy consumption of the UAV-enabled MEC system under the binary offloading mode.

Even though there are a lot of studies regarding the UAV-enabled MEC system, we find that the previous studies do not focus on the energy efficiency problem of the UAV. The energy efficient problem of the UAV is important as the battery capacity is limited, especially for the rotary-wing UAVs. Hence, in this paper, we study the energy efficiency maximization problem of the UAV while providing computation offloading services.

### 1.2. Contribution

In this paper, we are mainly concerned with the design of the energy efficient scheme of the UAV while providing on-demand offloading services in the hotspot areas or in the emergency scenarios under the partial offloading mode. With the goal of maximization of the energy efficiency of the UAV, the bits allocation in each time slot and the trajectory of the UAV are jointly optimized. Besides the constraints of energy capability of the UAV, the data causality and the velocity limitation are also taken into consideration for the optimization problem. The following part summarizes the main contributions of this paper.

- The energy efficiency maximization problem of the UAV while providing computation offloading services is formulated. Taking the constraints of the battery capacity of the UAV, the data causality, the speed limitation of the UAV into consideration, the trajectory of the UAV trajectory and bits allocation for transmitting and computing in each time slot are jointly optimized.
- A successive convex approximation (SCA)based alternating algorithm is presented to deal with the non-convex optimization problem. The energy efficiency maximization problem is non-convex due to the objectives and constraints. Additionally, the duality of the optimization variables also makes it hard to solve the optimization problem. By applying the proposed SCA-based alternating algorithm, the non-convex problem can be solved.
- The performance of the proposed energy-efficient design of the UAV is evaluated by the simulation results. With the simulation results, the proposed energy-efficient scheme design is verified to outperforms in many aspects when compared with two benchmark schemes. Furthermore, the effects of transmitting power and task deadline are also compared on the behavior of the proposed energy efficient design.

The rest of the paper is structured, as follows. Section 2 succinctly presents the system model and formulates the optimization problem. Section 3 introduces a SCA-based alternating algorithm to deal with the optimization problem. Then, in Section 4, the simulation results are depicted demonstrating the superiority of the proposed energy-efficient design compared with the benchmark schemes. Additionally, the performance of the proposed energy-efficient design is also displayed with the simulation results. Finally, the conclusions are drawn in Section 5.

## 2. System Model and Problem Formulation

**Figure 1:**
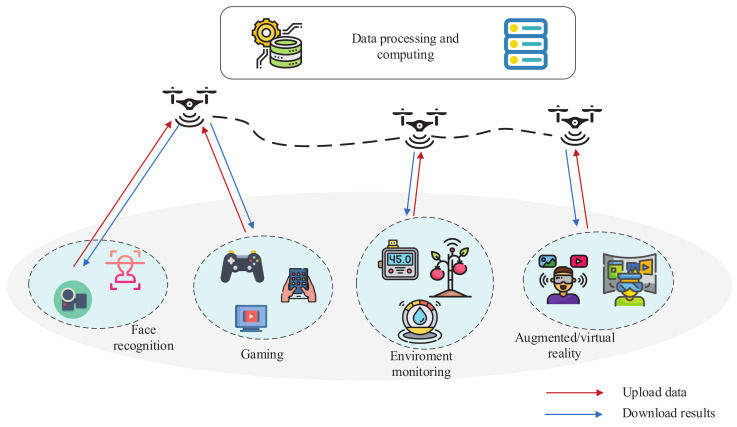
Unmanned aerial vehicles (UAV)-enabled mobile edge computing (MEC) system.

Partial offloading mode and binary offloading mode are two general offloading modes [8]. In binary offloading mode, the task is regarded as a whole part and it can not be partitioned in binary offloading mode, which should processed locally or transferred to the MEC server entirely. In the partial offloading mode, the tasks can be split into two parts: the local computing part and the offloading part. The partial offloading mode is considered to effectively adjust the number of uploading data of ground users with the goal of maximizing the energy efficiency of the UAV. It is assumed that one UAV and *K* ground users expressed as K≜{1,2,…,K} constitute the UAV-enabled MEC system. The UAV-enabled MEC system is depicted in Figure 1. The ground users upload the data, such as the face regonition data, gaming data, environment monitoring data, augmented/virtual reality data, and so on, in order to the UAV. After the UAV receiving the data from the ground users, it starts to process and compute the data. Subsequently, the UAV downloads the compuing results, such as the results of identification, rendering and stitching of video streams, environment analysis, and so on, to the ground users.

### 2.1. System Model

The task of usr *k* can be notated as $A_{k}\left(I_{k},C_{k},O_{k},\tau_{k}\right)$. $I_{k}$ represents the sum of the task bits of user *k*. $C_{k}$ denotes the required number of CPU cycles to compute one input bit for user *k*. $O_{k}$ is the ratio of the number of output bits to the number of input bits for user *k*. $\tau_{k}$ denotes the task deadline of user *k*. Besides, *K* ground users are assumed to be distributed as poisson point process (PPP). The offloading factor of the user *k* is $a_{k}$, which means that the user *k* sends $a_{k}I_{k}$ ($0\leq a_{k}\leq 1$) bits to the UAV for offloading and process $\left(1-a_{k}\right)I_{k}$ bits locally.

**Figure 2:**
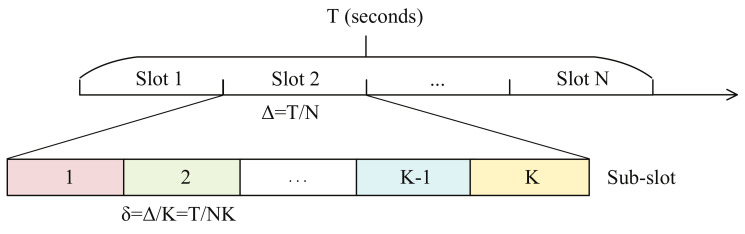
The slots and sub-slots division.

Time division multiple access (TDMA) mode is assumed to be the communication mode in the UAV-enabled MEC system in order to avoid the transmission interference. The time duration of the UAV-enabled MEC system *T* is assumed to be the shortest $\tau_{k}$ of all users. The time duration is assumed to be discretized into *N* slots, and the duration of each slot is $\Delta =\frac{T}{N}$. Besides, each slot is assumed to be discretized into *K* sub-slots, and the duration of each sub-slot is $\delta =\frac{\Delta }{K}=\frac{T}{NK}$. The user *k* only transmits the data which needs to be computed at the UAV in the *k*th sub-slot in each slot. Similarly, the UAV only sends the computing results to the user *k* in *k*th sub-slot in each slot. The slots and sub-slots are depicted as Figure 2. In this paper, we apply a three-dimensional Euclidean coordinate, where the units are meters, in order to reduce the loss of generality. The coordinate of ground user *k* is denoted as $q_{k}=\left(x_{k},y_{k},0\right)$. The location of the UAV in *n*th slot is denoted as $q_{u}\left[n\right]=\left(x_{u}\left[n\right],y_{u}\left[n\right],h\right)$. It is supposed that the communication channels between the UAV and the ground users are dominated by the line of sight (LoS) channel. Additionally, it is assumed that the Dopplor effect that is caused by the mobility of the UAV is compensated [22]. Consequently, the channel between the UAV and ground user *k* in *n*th slot follows the free-space path loss model, which is expressed as

(1) $$h_{k}\left(n\right)=\frac{g_{0}}{h^{2}+{∥q_{u}\left[n\right]-q\left[k\right]∥}^{2}},$$

where $g_{0}$ denotes the received power at the reference distance 1 m. The instantaneous channel capacity between the ground user *k* and the UAV in *n*th slot measured by bits/second is expressed as

(2) $$R_{k}\left[n\right]=B\log_{2}\left(1+\frac{pg_{0}}{(h^{2}+∥q_{u}\left[n\right]-q\left[k\right]{∥}^{2})\sigma^{2}}\right),$$

where *B* denotes the channel bandwidth between the UAV and the ground user, $\sigma^{2}$ represents the noise power at the receiver, *p* denotes the transmit power of ground users or the UAV. In this paper, it is assumed that the transmitting power of ground users and the UAV maintain the same value *P*. From (2), it can be observed that altering the two-dimensional (2D) location of the UAV and altering the altitude of the UAV have the same effects on the channel capacity. Consequently, it is assumed that the UAV flies at a certain altitude *H* in this paper. With the fixed altitude, Equation (2) can be expressed as

(3) $$R_{k}\left[n\right]=B\log_{2}\left(1+\frac{Ph_{0}}{(H^{2}+∥q_{u}\left[n\right]-q\left[k\right]{∥}^{2})\sigma^{2}}\right).$$

The total energy consumption of the UAV is composed of three parts: the propulsion energy consumption $E_{P}$, the computing energy consumption $E_{C}$, and the transmitting energy consumption $E_{T}$, which is given as Equation (4).

(4) $$E_{U}=E_{P}+E_{C}+E_{T}.$$

#### The Energy Consumption of the UAV

- Propulsion Energy Consumption

The propulsion energy consumption model of the UAV is in reference to the propulsion model proposed in [46,47,48]. For a rotary-wing UAV with speed *V*, the propulsion power consumption can be modeled as

(5) $$P\left(V\right)=\underset{blade\;profile}{\underbrace{P_{0}\left(1+\frac{3V^{2}}{U_{tip}^{2}}\right)}}+\underset{induced}{\underbrace{P_{i}{\left(\sqrt{1+\frac{V^{4}}{4v_{0}^{4}}}-\frac{V^{2}}{2v_{0}^{2}}\right)}^{\frac{1}{2}}}}+\underset{parasite}{\underbrace{\frac{1}{2}d_{0}\rho sAV^{3}}},$$

where $P_{0}$ and $P_{i}$ are blade profile power and induced power in hovering status, $U_{tip}$ denotes the tip speed of the rotor blade, $v_{0}$ denotes the mean rotor induced velocity in forwarding flight, $d_{0}$ and *s* represent the fuselage drag ratio and rotor solidity, respectively, and ρ and *A* denote the air density and rotor disk area, respectively.

The absolute value of the UAV displacement in *n*th slot divided by the time period of each slot Δ denotes the velocity of the UAV in *n*th slot, which can expressed as

(6) $$v\left[n\right]=\frac{q_{u}\left[n+1\right]-q_{u}\left[n\right]}{\Delta }.$$

Besides, the absolute value of the velocity of the UAV in each slot should not exceed the allowed value, which is shown as

(7) $$∥v\left[n\right]∥=\frac{∥q_{u}\left[n+1\right]-q_{u}\left[n\right]∥}{\Delta }≜\frac{D[n]}{\Delta }\leq V_{max},$$

where $V_{max}$ denotes the allowed velocity value of the UAV. By introducing Equation (7) into Equation (5), the energy consumed by propulsion in *n*th slot is derived as

(8) $$E_{P}\left[n\right]=P_{0}\left(\Delta +\frac{3D{\left[n\right]}^{2}}{\Delta U_{tip}^{2}}\right)+P_{i}{\left(\sqrt{\Delta^{4}+\frac{D{\left[n\right]}^{4}}{4v_{0}^{4}}}-\frac{D{\left[n\right]}^{2}}{2v_{0}^{2}}\right)}^{\frac{1}{2}}+\frac{1}{2}d_{0}\rho sA\frac{D{\left[n\right]}^{3}}{\Delta^{2}}.$$

Thus, within the time period *T*, the propulsion energy consumption is derived as

(9) $$E_{P}=P_{0}\sum_{n=1}^{N}\left(\Delta +\frac{3D{\left[n\right]}^{2}}{\Delta U_{tip}^{2}}\right)+P_{i}\sum_{n=1}^{N}{\left(\sqrt{\Delta^{4}+\frac{D{\left[n\right]}^{4}}{4v_{0}^{4}}}-\frac{D{\left[n\right]}^{2}}{2v_{0}^{2}}\right)}^{\frac{1}{2}}+\sum_{n=1}^{N}\frac{1}{2}d_{0}\rho sA\frac{D{\left[n\right]}^{3}}{\Delta^{2}}$$

•  Computing Energy Consumption

The required CPU frequency of the UAV to compute the tasks of the user *k* in *n*th slot is relevant to the number of computing bits in *n*th slot, which is denoted as

(10) $$f_{u,k}\left[n\right]=\frac{I_{k}^{c}\left[n\right]C_{k}}{\Delta },$$

where $I_{k}^{c}\left[n\right]$ represents the number of computing bits at the UAV for user *k* in *n*th slot. The computing energy consumption of the UAV in *n*th slot changes along with the computing frequency in *n*th slot $f_{u,k}\left[n\right]$. Hence, the computing energy consumption for user *k* at the UAV in *n*th slot is derived as

(11) $$E_{k}^{C}\left[n\right]=\Delta \gamma_{u}{\left(f_{u,k}\left[n\right]\right)}^{3}=\frac{\gamma_{u}C_{k}^{3}{\left(I_{k}^{c}\left[n\right]\right)}^{3}}{\Delta^{2}},$$

where $\gamma_{u}$ is the effective switched capacitance of the CPU [38,40,49,50]. The computing energy consumption of the UAV during the period of *T* is expressed as

(12) $$E_{C}=\sum_{n=1}^{N}\sum_{k=1}^{K}\frac{\gamma_{u}C_{k}^{3}{\left(I_{k}^{c}\left[n\right]\right)}^{3}}{\Delta^{2}}.$$

•  Transmitting Energy Consumption

After the UAV processes, the tasks uploading by the ground users and the computing results are sent back to the ground users. The UAV downloads the results with the constant transmitting power *P*. Hence, the transmitting energy consumption of the UAV during the period of *T* is shown as

(13) $$E_{T}=PT.$$

It is assumed that the UAV flies autonomously to provide offloading services for ground users in this paper. Accordingly, the communication between the UAV and the ground station is assumed to be ignored in this paper.

### 2.2. Problem Formulation

In this paper, the energy efficiency of the UAV is defined as

(14) $$EE_{U}=\frac{\sum_{k=1}^{K}a_{k}I_{k}}{E_{U}},$$

where $a_{k}$ denotes the offloading proportion of the task of user *k* and $a_{k}I_{k}=\sum_{n=1}^{N-2}I_{k}^{u}\left[n\right]=\sum_{n=2}^{N-1}I_{k}^{c}\left[n\right]=\sum_{n=3}^{N}I_{k}^{d}\left[n\right]/O_{k}$. $I_{k}^{u}$ denotes the number of uploading bits of the user *k* in *n*th slot and $I_{k}^{d}$ is the number of the downloading bits from the UAV to user *k* in *n*th slot. In the optimization problem, the energy efficiency of the UAV is maximized, which can be formulated as

(15a) $$\begin{array}{cc} P1:\; & \max_{I_{k}^{u}\left[n\right],I_{k}^{c}\left[n\right],I_{k}^{d}\left[n\right],q_{u}\left[n\right]}EE_{U}, \end{array}$$

(15b) $$\begin{array}{cc} \; & s.t.\;E_{F}\leq \varepsilon , \end{array}$$

(15c) $$\begin{array}{cc} \; & \sum_{n=1}^{N-2}I_{k}^{u}\left[n\right]=\sum_{n=2}^{N-1}I_{k}^{c}\left[n\right]\leq I_{k} \end{array}$$

(15d) $$\begin{array}{cc} \; & O_{k}\sum_{n=2}^{N-1}I_{k}^{c}\left[n\right]=\sum_{n=3}^{N}I_{k}^{d}\left[n\right]\leq O_{k}I_{k}, \end{array}$$

(15e) $$\begin{array}{cc} \; & \sum_{i=1}^{n-1}I_{k}^{u}\left[i\right]\geq \sum_{i=2}^{n}I_{k}^{c}\left[i\right],\;n=2,3,\ldots ,N-1, \end{array}$$

(15f) $$\begin{array}{cc} \; & O_{k}\sum_{i=2}^{n}I_{k}^{c}\left[i\right]\geq \sum_{i=3}^{n+1}I_{k}^{d}\left[i\right],\;n=2,3,\ldots ,N, \end{array}$$

(15g) $$\begin{array}{cc} \; & I_{k}^{u}\left[N-1\right]=I_{k}^{u}\left[N\right]=0, \end{array}$$

(15h) $$\begin{array}{cc} \; & I_{k}^{c}\left[1\right]=I_{k}^{c}\left[N\right]=0, \end{array}$$

(15i) $$\begin{array}{cc} \; & I_{k}^{d}\left[1\right]=I_{k}^{d}\left[2\right]=0, \end{array}$$

(15j) $$\begin{array}{cc} \; & I_{k}^{u}\left[n\right],I_{k}^{c}\left[n\right],I_{k}^{d}\left[n\right]\geq 0,for\;k\in K\;and\;n\in N, \end{array}$$

(15k) $$\begin{array}{cc} \; & I_{k}^{u}\left[n\right],I_{k}^{d}\left[n\right]\leq R_{k}\left[n\right]\delta , \end{array}$$

(15l) $$\begin{array}{cc} \; & q_{u}\left[1\right]=q_{u}^{S},q_{u}\left[n\right]=q_{u}^{F}, \end{array}$$

(15m) $$\begin{array}{cc} \; & ∥v\left[n\right]∥\leq V_{max},\;for\;n\in N, \end{array}$$

where ε represents the battery storage capacity of the UAV, N≜{1,2,…,N}, $q_{u}^{S}=\left(x_{1},y_{1},H\right)$, and $q_{u}^{F}=\left(x_{K},y_{K},H\right)$. Equation (15b) ensures that the UAV can complete the offloading services fot ground users with the battery capacity limitation of the UAV. Equations (15c) and (15d) ensure that all of the offloading tasks can be computed and the processing results can be sent to the ground users within the period of *T*. Additionally, it should be guaranteed that the total number of the offloading bits of the user *k* is supposed to be equal to the total number of bits for the task of the user *k*. Equations (15e)–(15i) guarantee the data causality of the offloading data, i.e., the UAV can only process the data after the data is uploaded to the UAV and the UAV can only download the computing results when the data have been processed by the UAV. Equation (15j) ensures the non-negativity of uploading bits, computing bits, and downloading bits in each slot. Equation (15k) ensures that the number of transmitting bits between user *k* and the UAV in each sub-slot is less than the channel capacity between the UAV and user *k*. Equation (15l) guarantees that the initial position of the UAV is over user 1 and the termination position of the UAV is over usr *K*. Equation (15m) guarantees that the speed of the UAV in each slot should be lower than the allowed maximal velocity of the UAV.

## 3. Algorithm Design

The energy efficiency maximization problem P1 is non-convex because of the non-convex objective function Equation (15a), non-convex constraints Equation (15b), Equation (15k), and Equation (15m). Additionally, the duality of the optimization variables also enhances the difficulty. To solve the non-convex optimization problem, we propose a SCA-based two-stage alternating algorithm in this paper. There are two steps in the proposed SCA-based two-stage alternating algorithm. In the first step, under the given trajectory of the UAV, the bits allocation is optimized. In the second step, when the bit allocation is given, the trajectory of the UAV is optimized. Subsequently, the two steps iterate successively.

### 3.1. Tasks Bits Allocation

When the trajectory of the UAV is given, P1 is converted to

(16a) $$\begin{array}{cc} \; & P2:\;\max_{I_{k}^{u}\left[n\right],I_{k}^{c}\left[n\right],I_{k}^{d}\left[n\right]}EE_{U}, \end{array}$$

(16b) $$\begin{array}{cc} \; & s.t.\;E_{F}\leq \varepsilon , \end{array}$$

(16c) $$\begin{array}{cc} \; & \sum_{n=1}^{N-2}I_{k}^{u}\left[n\right]=\sum_{n=2}^{N-1}I_{k}^{c}\left[n\right]\leq I_{k} \end{array}$$

(16d) $$\begin{array}{cc} \; & O_{k}\sum_{n=2}^{N-1}I_{k}^{c}\left[n\right]=\sum_{n=3}^{N}I_{k}^{d}\left[n\right]\leq O_{k}I_{k}, \end{array}$$

(16e) $$\begin{array}{cc} \; & \sum_{i=1}^{n-1}I_{k}^{u}\left[i\right]\geq \sum_{i=2}^{n}I_{k}^{c}\left[i\right],\;n=2,3,\ldots ,N-1, \end{array}$$

(16f) $$\begin{array}{cc} \; & O_{k}\sum_{i=2}^{n}I_{k}^{c}\left[i\right]\geq \sum_{i=3}^{n+1}I_{k}^{d}\left[i\right],\;n=2,3,\ldots ,N, \end{array}$$

(16g) $$\begin{array}{cc} \; & I_{k}^{u}\left[N-1\right]=I_{k}^{u}\left[N\right]=0, \end{array}$$

(16h) $$\begin{array}{cc} \; & I_{k}^{c}\left[1\right]=I_{k}^{c}\left[N\right]=0, \end{array}$$

(16i) $$\begin{array}{cc} \; & I_{k}^{d}\left[1\right]=I_{k}^{d}\left[2\right]=0, \end{array}$$

(16j) $$\begin{array}{cc} \; & I_{k}^{u}\left[n\right],I_{k}^{c}\left[n\right],I_{k}^{d}\left[n\right]\geq 0,for\;k\in K\;and\;n\in N, \end{array}$$

(16k) $$\begin{array}{cc} \; & I_{k}^{u}\left[n\right],I_{k}^{d}\left[n\right]\leq R_{k}\left[n\right]\delta . \end{array}$$

P2 can be further transformed as

(17a) $$\begin{array}{cc} \; & P2.1:\;\min_{I_{k}^{u}\left[n\right],I_{k}^{c}\left[n\right],I_{k}^{d}\left[n\right]}EE_{U}^{\prime }, \end{array}$$

(17b) $$\begin{array}{cc} \; & s.t.\;E_{F}\leq \varepsilon , \end{array}$$

(17c) $$\begin{array}{cc} \; & \sum_{n=1}^{N-2}I_{k}^{u}\left[n\right]=\sum_{n=2}^{N-1}I_{k}^{c}\left[n\right]\leq I_{k} \end{array}$$

(17d) $$\begin{array}{cc} \; & O_{k}\sum_{n=2}^{N-1}I_{k}^{c}\left[n\right]=\sum_{n=3}^{N}I_{k}^{d}\left[n\right]\leq O_{k}I_{k}, \end{array}$$

(17e) $$\begin{array}{cc} \; & \sum_{i=1}^{n-1}I_{k}^{u}\left[i\right]\geq \sum_{i=2}^{n}I_{k}^{c}\left[i\right],\;n=2,3,\ldots ,N-1, \end{array}$$

(17f) $$\begin{array}{cc} \; & O_{k}\sum_{i=2}^{n}I_{k}^{c}\left[i\right]\geq \sum_{i=3}^{n+1}I_{k}^{d}\left[i\right],\;n=2,3,\ldots ,N, \end{array}$$

(17g) $$\begin{array}{cc} \; & I_{k}^{u}\left[N-1\right]=I_{k}^{u}\left[N\right]=0, \end{array}$$

(17h) $$\begin{array}{cc} \; & I_{k}^{c}\left[1\right]=I_{k}^{c}\left[N\right]=0, \end{array}$$

(17i) $$\begin{array}{cc} \; & I_{k}^{d}\left[1\right]=I_{k}^{d}\left[2\right]=0, \end{array}$$

(17j) $$\begin{array}{cc} \; & I_{k}^{u}\left[n\right],I_{k}^{c}\left[n\right],I_{k}^{d}\left[n\right]\geq 0,for\;k\in K\;and\;n\in N, \end{array}$$

(17k) $$\begin{array}{cc} \; & I_{k}^{u}\left[n\right],I_{k}^{d}\left[n\right]\leq R_{k}\left[n\right]\delta , \end{array}$$

where $EE_{U}^{\prime }=\frac{1}{EE_{U}}=\frac{E_{U}}{\sum_{k=1}^{K}a_{k}I_{k}}$. The objective function of P2.1 can be further expressed as

(18) $$\frac{E_{F}+E_{T}+E_{C}}{\sum_{k=1}^{K}\sum_{n=1}^{N}I_{k}^{c}\left[n\right]}=\frac{E_{F}+E_{T}}{\sum_{k=1}^{K}\sum_{n=1}^{N}I_{k}^{c}\left[n\right]}+\frac{\sum_{n=1}^{N}\sum_{k=1}^{K}\gamma_{u}C_{k}^{3}{\left(I_{k}\left[n\right]\right)}^{3}/\Delta^{2}}{\sum_{n=1}^{N}\sum_{k=1}^{k}I_{k}^{c}\left[n\right]},$$

where $E_{F}$ and $E_{T}$ are derived by Equation (9) and (13). It can be easily observed that the objective of P2.1 is non-convex because of the second part of Equation (18). The second part of Equation (18) is upper-bounded by

(19) $$\frac{\sum_{n=1}^{N}\sum_{k=1}^{K}\gamma_{u}C_{k}^{3}{\left(I_{k}\left[n\right]\right)}^{3}}{\Delta^{2}\sum_{n=1}^{N}\sum_{k=1}^{k}I_{k}^{c}\left[n\right]}=\frac{\gamma_{u}C_{k}^{3}}{\Delta^{2}}\sum_{n=1}^{N}\sum_{k=1}^{K}\frac{{\left(I_{k}^{c}\left[n\right]\right)}^{3}}{\sum_{n=1}^{N}\sum_{k=1}^{k}I_{k}^{c}\left[n\right]}\leq \frac{\gamma_{u}C_{k}^{3}}{\Delta^{2}}\sum_{n=1}^{N}\sum_{k=1}^{K}{\left(I_{k}^{c}\left[n\right]\right)}^{2}≜EE_{C,ub}^{\prime }.$$

Hence, P2.1 can be approximately solved by minimizing its upper bound as

(20a) $$\begin{array}{cc} \; & P2.2:\;\min_{I_{k}^{u}\left[n\right],I_{k}^{c}\left[n\right],I_{k}^{d}\left[n\right]}\frac{E_{F}+E_{T}}{\sum_{k=1}^{K}\sum_{n=1}^{N}I_{k}^{c}\left[n\right]}+EE_{C,ub}^{\prime }, \end{array}$$

(20b) $$\begin{array}{cc} \; & s.t.\;E_{F}\leq \varepsilon , \end{array}$$

(20c) $$\begin{array}{cc} \; & \sum_{n=1}^{N-2}I_{k}^{u}\left[n\right]=\sum_{n=2}^{N-1}I_{k}^{c}\left[n\right]\leq I_{k} \end{array}$$

(20d) $$\begin{array}{cc} \; & O_{k}\sum_{n=2}^{N-1}I_{k}^{c}\left[n\right]=\sum_{n=3}^{N}I_{k}^{d}\left[n\right]\leq O_{k}I_{k}, \end{array}$$

(20e) $$\begin{array}{cc} \; & \sum_{i=1}^{n-1}I_{k}^{u}\left[i\right]\geq \sum_{i=2}^{n}I_{k}^{c}\left[i\right],\;n=2,3,\ldots ,N-1, \end{array}$$

(20f) $$\begin{array}{cc} \; & O_{k}\sum_{i=2}^{n}I_{k}^{c}\left[i\right]\geq \sum_{i=3}^{n+1}I_{k}^{d}\left[i\right],\;n=2,3,\ldots ,N, \end{array}$$

(20g) $$\begin{array}{cc} \; & I_{k}^{u}\left[N-1\right]=I_{k}^{u}\left[N\right]=0, \end{array}$$

(20h) $$\begin{array}{cc} \; & I_{k}^{c}\left[1\right]=I_{k}^{c}\left[N\right]=0, \end{array}$$

(20i) $$\begin{array}{cc} \; & I_{k}^{d}\left[1\right]=I_{k}^{d}\left[2\right]=0, \end{array}$$

(20j) $$\begin{array}{cc} \; & I_{k}^{u}\left[n\right],I_{k}^{c}\left[n\right],I_{k}^{d}\left[n\right]\geq 0,for\;k\in K\;and\;n\in N, \end{array}$$

(20k) $$\begin{array}{cc} \; & I_{k}^{u}\left[n\right],I_{k}^{d}\left[n\right]\leq R_{k}\left[n\right]\delta . \end{array}$$

It can be simply observed that P2.1 is convex and the convex problem can be solved with CVX [51].

### 3.2. Trajectory Design

When the bits allocation $I_{k}^{u}\left[n\right],I_{k}^{c}\left[n\right]$, and $I_{k}^{d}\left[n\right]$ are given, P1 can be transformed as

(21a) $$\begin{array}{cc} \; & P3\;\min_{q_{u}\left[n\right]}EE_{U}^{\prime }, \end{array}$$

(21b) $$\begin{array}{cc} \; & s.t.\;E_{F}\leq \varepsilon , \end{array}$$

(21c) $$\begin{array}{cc} \; & I_{k}^{u}\left[n\right],I_{k}^{d}\left[n\right]\leq R_{k}\left[n\right]\delta , \end{array}$$

(21d) $$\begin{array}{cc} \; & q_{u}\left[1\right]=q_{u}^{S},q_{u}\left[n\right]=q_{u}^{F}, \end{array}$$

(21e) $$\begin{array}{cc} \; & ∥v\left[n\right]∥\leq V_{max},\;for\;n\in N, \end{array}$$

It can be seen that the objective of P3 and Equation (21b) are non-convex because of the term $P_{i}\sum_{n=1}^{N}{\left(\sqrt{\Delta^{4}+\frac{D{\left[n\right]}^{4}}{4v_{0}^{4}}}-\frac{D{\left[n\right]}^{2}}{2v_{0}^{2}}\right)}^{\frac{1}{2}}$ in $E_{U}^{F}$. Subsequently, the slack variable y[n] is brought in to solve the non-convex term shown as

(22) $$y\left[n\right]={\left(\sqrt{\Delta^{4}+\frac{D{\left[n\right]}^{4}}{4v_{0}^{4}}}-\frac{D{\left[n\right]}^{2}}{2v_{0}^{2}}\right)}^{\frac{1}{2}},\;n\in N,$$

which is equal to

(23) $$\frac{\Delta }{y{\left[n\right]}^{2}}=y{\left[n\right]}^{2}+\frac{D{\left[n\right]}^{2}}{v_{0}^{2}},\;n\in N.$$

Consequently, the objective function of P3 is shown as

(24) $$\begin{array}{cc} \; & EE_{U}^{\prime }=[P_{0}\sum_{n=1}^{N}\left(\Delta +\frac{3D{\left[n\right]}^{2}}{\Delta U_{tip}^{2}}\right)+P_{i}\sum_{n=1}^{N}y\left[n\right]+\sum_{n=1}^{N}\frac{1}{2}d_{0}\rho SA\frac{D{\left[n\right]}^{3}}{\Delta^{2}}+\\ \; & \sum_{n=1}^{N}\sum_{k=1}^{K}\frac{\gamma_{u}C_{k}^{3}{\left(I_{k}^{c}\left[n\right]\right)}^{3}}{\Delta^{2}}+PT]/\sum_{n=1}^{N}\sum_{k=1}^{K}I_{k}^{u}\left[n\right],\;n\in N,\;k\in K, \end{array}$$

taking the constraint Equation (23) into consideration. Subsequently, the optimization problem P3 can be expressed as

(25a) $$\begin{array}{cc} \; & P3.1\;\min_{q_{u}\left[n\right]}EE_{U}^{\prime }, \end{array}$$

(25b) $$\begin{array}{cc} \; & s.t.\;I_{k}^{u}\left[n\right],I_{k}^{d}\left[n\right]\leq R_{k}\left[n\right]\delta , \end{array}$$

(25c) $$\begin{array}{cc} \; & q_{u}\left[1\right]=q_{u}^{S},q_{u}\left[n\right]=q_{u}^{F}, \end{array}$$

(25d) $$\begin{array}{cc} \; & ∥v\left[n\right]∥\leq V_{max},\;for\;n\in N, \end{array}$$

(25e) $$\begin{array}{c} \begin{array}{cc} \; & P_{0}\sum_{n=1}^{N}\left(\Delta +\frac{3D{\left[n\right]}^{2}}{\Delta U_{tip}^{2}}\right)+P_{i}\sum_{n=1}^{N}y\left[n\right]+\sum_{n=1}^{N}\frac{1}{2}d_{0}\rho SA\frac{D{\left[n\right]}^{3}}{\Delta^{2}}\\ \; & +\frac{\sum_{n=1}^{N}\sum_{k=1}^{K}\gamma_{u}C_{k}^{3}{\left(I_{k}^{c}\left[n\right]\right)}^{3}}{\Delta }+PT\leq \varepsilon \end{array} \end{array}$$

(25f) $$\begin{array}{cc} \; & \frac{\Delta^{2}}{y{\left[n\right]}^{2}}\leq y{\left[n\right]}^{2}+\frac{D{\left[n\right]}^{2}}{v_{0}^{2}}, \end{array}$$

(25g) $$\begin{array}{cc} \; & y[n]\geq 0,\;n\in N. \end{array}$$

The equality in Equation (25f) is maintained at the optimal point of P3.1. It can be easily observed that P3.1 is still non-convex because of the non-convex constraints Equations (25b) and (25f). The right hand side of Equation (25b) is non-concave in regard to $q_{u}\left[n\right]$. Besides, it is observed that the left hand side of Equation (25f) is a joint convex function in regard to y[n] and $q_{u}\left[n\right]$. Hence, constraints Equations (25b) and (25f) are non-convex. The SCA technique is illustrated in Theorems 1 and 2. By applying the SCA technique, the non-convex constraints Equations (25b) and (25f) can be approximately expressed by convex constraints.

**Theorem** **1.** *The global concave lower bound of the right hand side of Equation *(25b)* can be expressed as*

(26) $$\begin{array}{cc} \; & R_{k}\left[n\right]\geq B\log_{2}\left(1+\frac{Ph}{\sigma^{2}(H^{2}+∥q_{u}{\left[n\right]}^{\left(l\right)}-q_{k}{∥}^{2})}\right)-\\ \; & \frac{(∥q_{u}\left[n\right]-q_{k}{∥}^{2}-∥q_{u}{\left[n\right]}^{\left(l\right)}-q_{k}{∥}^{2})PhB\log_{2}e}{(H^{2}+∥q_{u}{\left[n\right]}^{\left(l\right)}-q_{k}{∥}^{2})(Ph+\sigma^{2}H^{2}+\sigma^{2}∥q_{u}{\left[n\right]}^{\left(l\right)}-q_{k}{∥}^{2})}≜R_{k,lb}\left[n\right], \end{array}$$

*in which the equality is maintained when $q_{u}\left[n\right]=q_{u}{\left[n\right]}^{\left(l\right)}$.*

**Algorithm 1** SCA-based alternating algorithm for P1**Input:**$A_{k}$, *K*, *N*, Δ, δ, $q_{k}$, *P*, *B*, $\sigma^{2}$, $P_{i}$, $P_{0}$, $U_{tip}$, $v_{0}$, $d_{0}$, ρ, *s*, *A*, $V_{max}$, ε and tolerant threshold ξ, $\xi_{1}$;
1: 2:**Initialize:** Iterative number i=1, local point $q_{u}{\left[n\right]}^{\left(1\right)}$, $EE_{U}^{1}=0$;3: 4:
**repeat**
5: 6:    Solve **P2.2** by applying CVX under the given trajectory $q_{u}{\left[n\right]}^{\left(i\right)}$ and get the optimal bits allocation in each slot $I_{k}^{u*}\left[n\right]$, $I_{k}^{c*}\left[n\right]$ and $I_{k}^{d*}\left[n\right]$;7: 8:    Update the iterative number i=i+1;9: 10:    Let $I_{k}^{u,i}\left[n\right]=I_{k}^{u*}\left[n\right]$, $I_{k}^{c,i}\left[n\right]=I_{k}^{c*}\left[n\right]$ and $I_{k}^{d,i}\left[n\right]=I_{k}^{d*}\left[n\right]$;11: 12:    **repeat**13: 14:        **Initialize:** Iterative number l=1, local point $q_{u}{\left[n\right]}^{l}$ and $y{\left[n\right]}^{l}$, $EE_{U}^{\prime i,1}=0$;15: 16:        Solve **P3.1** by applying CVX under the given bits allocation in each slot $I_{k}^{u,i}\left[n\right]$$I_{k}^{c,i}\left[n\right]$$I_{k}^{d,i}\left[n\right]$ and get the optimal trajectory of the UAV $q_{u}^{*}\left[n\right]$;17: 18:        Update l=l+1;19: 20:        Let $q_{u}^{i,l}\left[n\right]=q_{u}^{*}\left[n\right]$;21: 22:        Get $EE_{U}^{\prime i,l}$;23: 24:    **until** $EE_{U}^{\prime i,l}-EE_{U}^{\prime i,l-1}\leq \xi_{1}$25: 26:    Obtain the energy efficiency of the UAV $EE_{U}^{i}$ by (14);27: 28:
**until** $EE_{U}^{i}-EE_{U}^{i-1}\leq \xi$
29: 30:Let $I_{k}^{u}\left[n\right]=I_{k}^{u,i}\left[n\right]$, $I_{k}^{c}\left[n\right]=I_{k}^{c,i}\left[n\right]$, $I_{k}^{d}\left[n\right]=I_{k}^{d,i}\left[n\right]$, $q_{u}\left[n\right]=q_{u}^{i,l}\left[n\right]$;31: **Output:**$I_{k}^{u}\left[n\right]$, $I_{k}^{c}\left[n\right]$, $I_{k}^{d}\left[n\right]$ and $q_{u}\left[n\right]$.32: 

**Proof.**  Let $f\left(z\right)=\log_{2}\left(1+\frac{B}{A+z}\right)$, z≥0. *A* and *B* are positive constants. It can be easily observed that f(z) is convex with regard to *z*. When considering the fact that the global lower bound of a convex function is its the first-order Taylor expansion, it can be obtained that $f\left(z\right)\geq f\left(z_{0}\right)+f^{\prime }\left(z_{0}\right)\left(z-z_{0}\right)$, where $f^{\prime }\left(z_{0}\right)=\frac{-B\log_{2}e}{\left(A+z_{0}\right)\left(B+A+z_{0}\right)}$. When $z_{0}=0$, it can be obtained that

(27) $$\log_{2}\left(1+\frac{B}{A+z}\right)\geq \log_{2}\left(1+\frac{B}{A}\right)-\frac{(\log_{2}e)Bz}{A(A+B)}.$$

Let $B=\frac{Ph}{\sigma^{2}}$, $A=H^{2}+{∥q_{u}{\left[n\right]}^{\left(l\right)}-q_{k}∥}^{2}$ and $z=∥q_{u}\left[n\right]-q_{k}{∥}^{2}-{∥q_{u}{\left[n\right]}^{\left(l\right)}-q_{k}∥}^{2}$, the following inequality is derived:

(28) $$\begin{array}{cc} \; & R_{k}\left[n\right]\geq B\log_{2}\left(1+\frac{Ph}{\sigma^{2}+(H^{2}+∥q_{u}{\left[n\right]}^{\left(l\right)}-q_{k}{∥}^{2})}\right)-\\ \; & \frac{\left(\log_{2}e\right)PhB(∥q_{u}\left[n\right]-q_{k}{∥}^{2}-∥q_{u}{\left[n\right]}^{\left(l\right)}-q_{k}{∥}^{2})}{(H^{2}+∥q_{u}{\left[n\right]}^{\left(l\right)}-q_{k}{∥}^{2})(Ph+\sigma^{2}H^{2}+\sigma^{2}∥q_{u}{\left[n\right]}^{\left(l\right)}-q_{k}{∥}^{2})}, \end{array}$$

where $q_{u}{\left[n\right]}^{\left(l\right)}$ is the local value of $q_{u}\left[n\right]$ at the *l*th iteration.  □

Furthermore, using the first-order Taylor expansion, the lower bound of right-hand side in Equation (25f) is obtained, as shown Theorem 2.

**Theorem** **2.** *The global concave lower bound of the right-hand side of Equation *(25f)* is derived as*

(29) $$\begin{array}{cc} \; & y{\left[n\right]}^{2}+\frac{D{\left[n\right]}^{2}}{v_{0}^{2}}\geq y{\left[n\right]}^{\left(l\right)}+2y{\left[n\right]}^{\left(l\right)}\left(y\left[n\right]-y{\left[n\right]}^{\left(l\right)}\right)-\frac{∥q_{u}{\left[n+1\right]}^{\left(l\right)}-q_{u}{\left[n\right]}^{\left(l\right)}{∥}^{2}}{v_{0}^{2}}+\\ \; & \frac{2}{v_{0}}(∥q_{u}{\left[n+1\right]}^{\left(l\right)}-q_{u}{\left[n\right]}^{\left(l\right)}∥)(∥q_{u}\left[n+1\right]-q_{u}\left[n\right]∥)≜Y_{n}^{\left(l\right)}\left(q_{u}\left[n\right]\right), \end{array}$$

*where $q_{u}{\left[n\right]}^{\left(l\right)}$ and $y{\left[n\right]}^{\left(l\right)}$ denote the local value of $q_{u}\left[n\right]$ and y[n] at the lth iteration, respectively.*

By applying Theorem 1 and Theorem 2, P3.1 can be converted as

(30a) $$\begin{array}{cc} \; & P3.1\;\min_{q_{u}\left[n\right],y\left[n\right]}EE_{U}^{\prime }, \end{array}$$

(30b) $$\begin{array}{cc} \; & s.t.\;q_{u}\left[1\right]=q_{u}^{S},q_{u}\left[n\right]=q_{u}^{F}, \end{array}$$

(30c) $$\begin{array}{cc} \; & ∥v\left[n\right]∥\leq V_{max},\;for\;n\in N, \end{array}$$

(30d) $$\begin{array}{c} \begin{array}{cc} \; & P_{0}\sum_{n=1}^{N}\left(\Delta +\frac{3D{\left[n\right]}^{2}}{\Delta U_{tip}^{2}}\right)+P_{i}\sum_{n=1}^{N}y\left[n\right]+\sum_{n=1}^{N}\frac{1}{2}d_{0}\rho SA\frac{D{\left[n\right]}^{3}}{\Delta^{2}}\\ \; & +\frac{\sum_{n=1}^{N}\sum_{k=1}^{K}\gamma_{u}C_{k}^{3}{\left(I_{k}^{c}\left[n\right]\right)}^{3}}{\Delta }+PT\leq \varepsilon \end{array} \end{array}$$

(30e) $$\begin{array}{cc} \; & I_{k}^{u}\left[n\right],I_{k}^{d}\left[n\right]\leq R_{k,lb}\left[n\right]\delta \end{array}$$

(30f) $$\begin{array}{cc} \; & \frac{\Delta^{2}}{y{\left[n\right]}^{2}}\leq Y_{n}^{\left(l\right)}\left(q_{u}\left[n\right]\right), \end{array}$$

(30g) $$\begin{array}{cc} \; & y[n]\geq 0,\;n\in N. \end{array}$$

Subsequently, the CVX solver can be applied to solve **P3.1** [51].

By successively iterating the aforementioned two steps, a SCA-based alternating algorithm is brought forward to tackle the energy efficiency maximization problem **P1**. The SCA-based alternating algorithm is illustrated in Algorithm 1.

## 4. Simulation Results

In this section, the simulation results are depicted, showing the performance of the proposed energy efficient design. At first, the superiority of the proposed design is testified by comparing with two benchmark schemes. Next, we explore how the energy efficiency of the UAV changes along with transmitting power and time constraint. The communication and computing parameters are set as: B=40 MHz, $\gamma_{u}=10^{-28}$, $\sigma^{2}=10^{-9}$ W, $h_{0}=-30$ dB, $O_{k}=0.5$, and $C_{k}=1500$ cycle/bits, which are based on the work in [38,40]. The parameters that are related to the propulsion of the UAV are set as: $P_{0}=79.9$ W, $P_{i}=88.63$ W, $U_{tip}=120$ m/s, $v_{0}=4.03$ m/s, $d_{0}=0.6$, $s=0.05\;m^{3}$, $\rho =1.225\;\mathrm{kg}/m^{3}$, and $A=0.503\;m^{2}$, which are based on the work in [47]. The fixed altitude of the UAV is assumed as H=20 m. The allowed maximal velocity of the UAV is assumed to be 15 m/s. The tolerance thresholds in the simulations are set as $10^{-3}$ to guarantee the convergence. The simulation parameters are shown in Table 1 [38,40,47].

**Figure 3:**
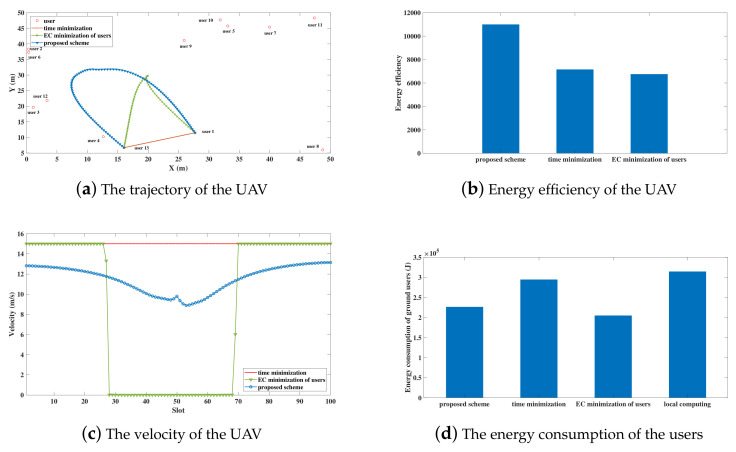
Performance comparison of 13 users under the proposed energy-efficient scheme and benchmark (T=5 s, P=0.2 W).

Firstly, we compare the energy efficiency of the UAV in the UAV-enabled MEC system with other benchmark schemes. The ground users follow the PPP distribution in 50 m × 50 m region. The transmitting power of ground users and the UAV are preset to be 0.2 W. The time period is assumed to be 5 s. The amount of task bits of each user follows the random distribution from $10^{8}-10^{9}$, which is assumed to be $I_{1}=1.3459\times 10^{8}$ bits, $I_{2}=8.0595\times 10^{8}$ bits, $I_{3}=5.2476\times 10^{8}$ bits, $I_{4}\;=\;9.4426\;\times \;10^{8}$ bits, $I_{5}=9.88348\times 10^{8}$ bits, $I_{6}=4.0989\times 10^{8}$ bits, $I_{7}=3.7119\times 10^{8}$ bits, $I_{8}=2.2685\times 10^{8}$ bits, $I_{9}=4.4603\times 10^{8}$ bits, $I_{10}=2.6622\times 10^{8}$ bits, $I_{11}=4.5910\times 10^{8}$ bits, $I_{12}=4.3291\times 10^{8}$ bits, $I_{13}=2.5962\times 10^{8}$ bits. In Figure 3, the trajectories of the UAV, energy efficiency of the UAV, the velocity of the UAV, and the energy consumption of ground users are shown under the same users distribution with the proposed energy efficient scheme and two benchmark schemes: (i) time minimization scheme and, (ii) energy consumption (EC) minimization of users scheme, as elaborated in [40]. In time minimization scheme, the UAV flies directly from user 1 to user *K* at the maximum velocity and try the best of the UAV to processes tasks. In EC minimization of users scheme, the total energy consumption of ground users, which consists of the communication energy consumption and the computing energy consumption, is minimized within the limited time period [40].

The trajectories of the UAV using the proposed energy-efficient scheme and two benchmark schemes are compared in Figure 3a. It can be observed that, in the proposed energy-efficient scheme, the UAV flies closer to ground users in order to provide better offloading services. Additionally, for the EC minimization scheme, the UAV went through a sharp turn to minimize the energy consumption of users, neglecting the energy consumption of the UAV. Figure 3c depicts the velocities of the UAV using three schemes, respectively. In the proposed energy efficient scheme, the UAV alters the velocity on the basis of the number of uploading bits, which leads to the increase of the energy efficiency of the UAV. For the time minimization scheme, UAV flies directly from the source user to the end user with the allowed maximal velocity. Additionally, for the EC minimization of users scheme, the velocity of UAV goes through a big changes as this scheme ignores the energy consumption of UAVs.

**Figure 4:**
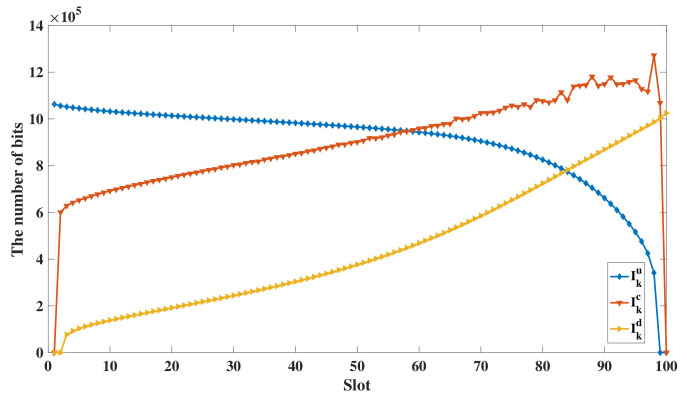
The bits allocation of user 1 under the proposed energy-efficient design of the UAV (T=5 s, P=0.2 W).

In Figure 3b, the energy efficiency of the UAV using three schemes are compared. It can be easily observed that the proposed scheme performs better than the two benchmark schemes in the aspect of energy-efficiency greatly. The energy efficiency of the proposed scheme, the time minimization scheme, and the EC minimization of users scheme are 11004, 7159.9 and 6757.7 separately, as shown in Figure 3b. In the time minimization scheme, the energy consumption enhancement of the UAV is caused by the maximum velocity of the UAV. In addition, the distance from the direct trajectory to ground users impacts the link connections. Hence, the decreasing number of receiving bits at the UAV leads to the deterioration of the energy efficiency. In EC minimization of users scheme, the ground users try the best to offload tasks to the UAV and the energy consumption of ground users is minimized. Besides, the energy consumption of the UAV is neglected, which results in the poor energy efficiency of the UAV. Figure 3d displays the energy consumption of the ground users while using the proposed energy-efficient scheme, two benchmark schemes, and a local computing scheme. In the local computing scheme, all pf the computation tasks are processed by the ground users locally under the time constraint *T*. It can be seen that the energy consumption of ground users using the other three schemes is lower than that of the local computing scheme. It is because that the ground users compute all of the tasks locally, which causes the high computing energy consumption. By comparing the energy consumption of users in the three schemes, the time minimization scheme performs the worst, while the EC minimization of users scheme performs the best. The EC minimization of users scheme has the lowest energy consumption of ground users because the objective is to minimize the energy consumption of ground users. In a nutshell, the proposed energy-efficient scheme is superior to other benchmark schemes prominently. Additionally, the lifetime of ground users using the proposed scheme is prolonged because the computing burden is relieved by the UAV. Secondly, Figure 4 shows the bits allocation in each slot of user 1 in Figure 3a. It can be seen that the amount of uploading bits decreases along with time for the proposed scheme. In contrast, the number of computing bits and downloading bits increase along with time. The phenomenon is because of the data causality, which means that the UAV can only process the tasks when the ground users finish the transmission of data and the computing results can only be transmitted to the ground users when the UAV complete data processing. What is more, to finish the offloading services in time, the number of computing bits and downloading bits exceed the number of uploading bits gradually. In addition, the number of downloading bits is relatively low because $O_{k}$ is lower than 1.

**Figure 5:**
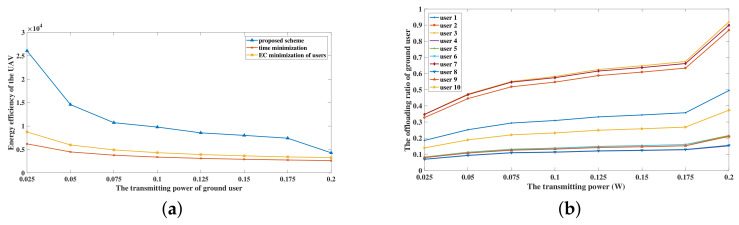
The performance of the UAV under different transmitting power. (**a**) Energy efficiency of the UAV with different transmitting power; (**b**) Offloading ratio of ground users with different transmitting power under the proposed scheme.

Figure 5 depicts the performance of the UAV by using different transmitting power *P*, which is interpreted as the transmitting power of ground users and the UAV. The time constraint is preset to be T = 5 s. Additionally, the ground users follow PPP distribution in 50 m × 50 m region. In Figure 5a, the effects of *P* on the energy efficiency of the UAV under the proposed scheme, the time minimization scheme and the EC minimization of users scheme are depicted. No matter how the value of *P* changes, the energy efficiency of the UAV using the proposed scheme is always higher than that of the other two benchmark schemes. Besides, the energy efficiency of the UAV using three schemes descends when the value of *P* grows. From Figure 5b, it is obviously observed that the offloading ratio of each user grows when the transmitting power increases by using the proposed scheme. Likewise, it can be deduced that the offloading ratio of ground users with the other two benchmark schemes also increase when the transmitting power grows. As the offloading ratio grows, the number of uploading bits increases and the computing burden is aggravated. Subsequently, the higher computing energy consumption is caused by the higher computing burden. Besides, the energy efficiency of the UAV is decreased because the energy consumption of the UAV has a more obvious effect than the number of input bits on the energy efficiency of the UAV. Hence, when the transmitting power is growing, the energy efficiency using three schemes is decreasing. Besides, it can be inferred that, when the transmitting power grows, the computation burden of ground users decreases because of the growth of the offloading ratio.

**Figure 6:**
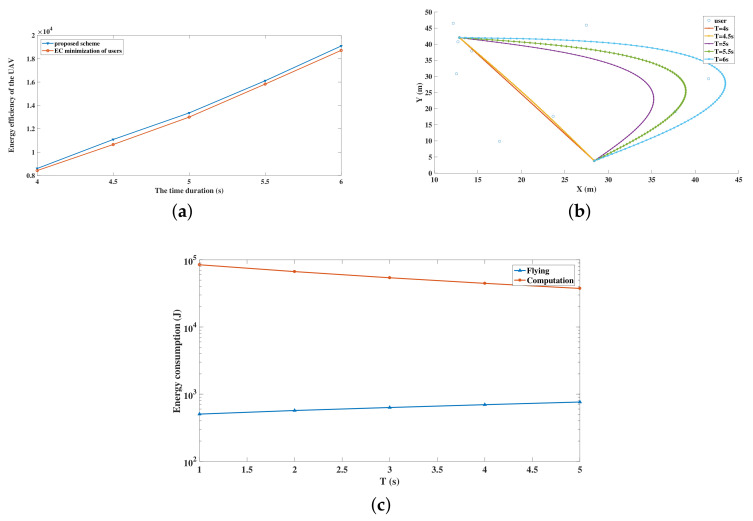
The performance of the UAV under different time constraints. (**a**) Energy efficiency of the UAV with different time constraints; (**b**) Trajectory of the UAV with different time constraints under the proposed scheme; (**c**) Energy consumption of the UAV with different time constraints under the proposed scheme.

At last, Figure 6 depicts the energy efficiency and trajectories of the UAV when considering different time constraints *T*, supposing that the P=0.2 W. The ground users follow the PPP distribution in 50 m × 50 m region. When applying the proposed scheme and the EC minimization of users scheme, the users follow the same distribution with the same offloading ratio as *T* varies. The energy efficiency of the UAV using the two schemes under different time constraints are shown in Figure 6a. It can be observed that no matter how *T* changes, the energy efficiency of the UAV using the proposed scheme maintains higher than that of the UAV using the EC minimization of users scheme. In addition, when *T* increases, the energy efficiency of the UAV under both schemes grows accordingly. This is because, when the time constraint becomes urgent, the UAV has to reduce the flight distance as a result of the velocity limitation, as plotted in Figure 6b. Besides, when the time constraint is urgent, the UAV has to complete the offloading tasks under the limited time duration and the computing burden is increased. Subsequently, the energy consumption of computing is increased, which causes the deterioration of the energy efficiency of the UAV. Even though the propulsion energy consumption increases because of the longer flight distance along with the growth of *T*, the computation burden is mitigated, owing to the growth of *T*, as shown in Figure 6c. What is more, it can be observed from Figure 6c that computing consumes more energy than flying. Hence, when the time constraint grows, the energy efficiency of the UAV rises, even if the energy consumption of flying increases.

## 5. Conclusions

This paper proposes an energy efficient scheme design of the UAV while providing task offloading services. The offloading services in the some specific areas with computation demand, such as hotspot areas and emergency areas, can be provided by the UAV thanks to the advantages of agility and easy deployment. The bits allocation of offloading data in each slot and the trajectory of the UAV are jointly optimized aiming at maximizing the energy efficiency of the UAV. Additionally, the constraints of the battery life of the UAV, the data causality, and the speed of the UAV are also taken into consideration. What is more, a SCA-based alternating algorithm is brought forward to tackle the non-convex energy efficiency maximization problem. Finally, it is verified that the proposed energy efficient scheme design outperforms other benchmark schemes with simulation results. Additionally, how the changes of transmitting power and time constraints impact the energy efficiency of the UAV is also discussed. Through this paper, we hope to provide some insights for future implementation and application design about the energy efficient UAV-enabled system, providing computation and connections for ground users and maintaining the high energy-efficiency. What is more, we will focus on the cooperative multiple UAVs based MEC system design to provide large-scale and energy-efficient offloading services for ground users in our future work.

## Figures and Tables

**Table 1:** Parameters setting.

Parameters	Description	Value
*B*	The communication channel bandwidth	40 Mhz
$O_{k}$	The proportion of the output bits to the inputs bits to for user *k*	0.5
$C_{k}$	The computation/intensity of user *k*	1500 cycles/bits
$\gamma_{u}$	The effective switched capacitance of the CPU of the UAV	$10^{-28}$
$\sigma^{2}$	Noise power at the receiver	$10^{-9}$ W
$h_{0}$	Received power at the reference distance 1m	−30 dB
*N*	The total number of slots	100
*H*	The altitude of the UAV	20 m
$P_{0}$	The blade profile power in hovering status	79.9 W
$P_{i}$	The induced power in hovering status	88.63 W
$U_{tip}$	The tip speed of the rotor speed	120 m/s
$v_{0}$	The mean rotor induced velocity in hovering status	4.03 m/s
$d_{0}$	The fuselage drag ratio	0.6
*s*	The rotor solidity	$0.05\;m^{3}$
ρ	The air density	$1.225\;\mathrm{kg}/m^{3}$
*A*	The rotor disk area	$0.503\;m^{2}$
$V_{max}$	The allowed maximal velocity of the UAV	15 m/s
ε	The battery storage capacity of the UAV	$5\times 10^{5}$ J
$\xi ,\xi_{1}$	The tolerance threshold	$10^{-3}$
